# Connective tissue growth factor mediates TGF-β1-induced low-grade serous ovarian tumor cell apoptosis

**DOI:** 10.18632/oncotarget.19626

**Published:** 2017-07-27

**Authors:** Jung-Chien Cheng, Hsun-Ming Chang, Peter C.K. Leung

**Affiliations:** ^1^ Department of Obstetrics and Gynaecology, BC Children’s Hospital Research Institute, University of British Columbia, Vancouver, British Columbia, V5Z 4H4, Canada

**Keywords:** apoptosis, CTGF, low-grade serous carcinoma, ovarian cancer, TGF-β1

## Abstract

Ovarian low-grade serous carcinoma (LGSC) is a rare disease and is now considered to be a distinct entity from high-grade serous carcinoma (HGSC), which is the most common and malignant form of epithelial ovarian cancer. Connective tissue growth factor (CTGF) is a secreted matricellular protein that has been shown to modulate many biological functions by interacting with multiple molecules in the microenvironment. Increasing evidence indicates that aberrant expression of CTGF is associated with cancer development and progression. Transforming growth factor-β1 (TGF-β1) is a well-known molecule that can strongly up-regulate CTGF expression in different types of normal and cancer cells. Our previous study demonstrated that TGF-β1 induces apoptosis of LGSC cells. However, the effect of TGF-β1 on CTGF expression in LGSC needs to be defined. In addition, whether CTGF mediates TGF-β1-induced LGSC cell apoptosis remains unknown. In the present study, we show that TGF-β1 treatment up-regulates CTGF expression by activating SMAD3 signaling in two human LGSC cell lines. Additionally, siRNA-mediated CTGF knockdown attenuates TGF-β1-induced cell apoptosis. Moreover, our results show that the inhibitory effect of the CTGF knockdown on TGF-β1-induced cell apoptosis is mediated by down-regulating SMAD3 expression. This study demonstrates an important role for CTGF in mediating the pro-apoptotic effects of TGF-β1 on LGCS.

## INTRODUCTION

Connective tissue growth factor (CTGF), also known as CCN2, is a secreted matricellular protein that belongs to the CCN protein family [1]. The members of the CCN protein family do not have structural roles, but they can regulate various cellular functions in response to different environmental stimuli by modulating the interactions between the cell and extracellular matrix [2, 3]. CTGF is the most studied member of the CCN protein family and was originally identified in the conditioned media of human umbilical vein endothelial cells as a platelet-derived growth factor-inducible immediate early gene [4]. To date, multiple biological functions of CTGF have been discovered in both physiological and pathological conditions [5].

Ovarian cancer is the most lethal of all gynecologic malignancies, and more than 90% of ovarian cancers are of epithelial origin [6]. The majority of epithelial ovarian cancers are of the serous subtype. Recently, it has been shown that serous ovarian carcinomas can be categorized into two groups that are designated low-grade serous carcinoma (LGSC) and high-grade serous carcinoma (HGSC) according to clinical, pathological, and molecular genetic studies [7]. LGSC is rare and is thought to develop from serous borderline ovarian tumors (SBOT). In contrast, HGSC, the most common form of epithelial ovarian cancer, is usually associated with aggressive clinical features and has been considered to rise from the ovarian surface epithelium (OSE) or from serous tubal intra-epithelial carcinomas in the fallopian tube [7, 8]. In the past few decades, researchers were mainly focused on the biology of HGSC, while LGSC has received little attention. It has been shown that relatively a poor prognosis, responsiveness to conventional chemotherapy and survival are observed when SBOT progresses to LGSC [9, 10]. Therefore, developments of reliable diagnostic and effective therapeutic methods will significantly improve the survival rate of patients with LGSC.

A few *in vitro* studies have demonstrated that CTGF promotes HGSC cell migration, growth and peritoneal adhesion [11, 12]. In addition, immunohistochemical studies reveal that the expression levels of CTGF are correlated to HGSC stage and negatively associated with poor survival [12, 13]. Our previous studies have shown that treatment with transforming growth factor-β1 (TGF-β1) or CD40 ligand can induce LGSC cell death [14, 15]. Interestingly, TGF-β1 has been identified as a strong molecule that can up-regulate CTGF expression in many types of normal and cancer cells derived from different organs including the ovary [3, 5, 16, 17]. However, to date, the effect of TGF-β1 on CTGF expression in LGSC needs to be defined. Furthermore, if TGF-β1 does up-regulate CTGF expression, whether CTGF mediates TGF-β1-induced LGSC cell death remains unknown. In this study, our results show that treatment with TGF-β1 up-regulated CTGF expression in two human LGSC cell lines through activation of the SMAD3 signaling pathway. In addition, siRNA-mediated CTGF knockdown attenuated TGF-β1-induced up-regulation of cleaved caspase-3 and cell apoptosis. Moreover, we find that the knockdown of CTGF attenuated TGF-β1 signaling by down-regulating SMAD3 expression.

## RESULTS

### TGF-β1 up-regulates CTGF expression in human LGSC cells

**Figure 1:**
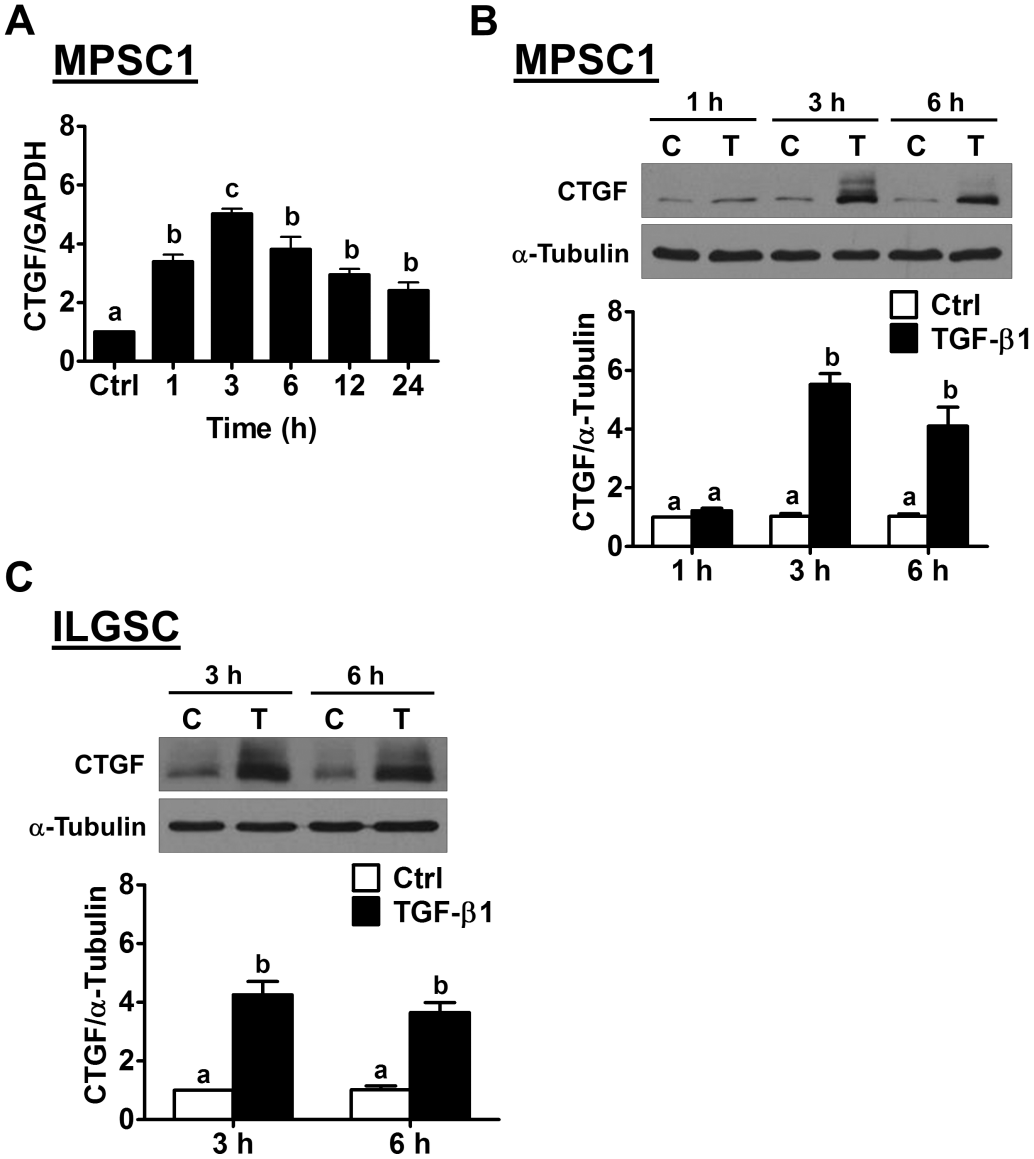
TGF-β1 up-regulates CTGF expression in human LGSC cells **(A)**, MPSC1 cells were treated with vehicle control (Ctrl) or 10 ng/mL TGF-β1, and the mRNA levels of CTGF were analyzed at different time points by RT-qPCR. The level of CTGF mRNA at each time point was normalized to the GAPDH mRNA level at the same time point. **(B)**, MPSC1 cells were treated with vehicle control (Ctrl or C) or 10 ng/mL TGF-β1 (T) for 1, 3 and 6 h, and the protein levels of CTGF were examined by western blot. **(C)**, ILGSC cells were treated with vehicle control (Ctrl or C) or 10 ng/mL TGF-β1 (T) for 3 and 6 h, and the protein levels of CTGF were examined by western blot. The results are expressed as the mean ± SEM of at least three independent experiments. Values without a common letter were significantly different (*p*<0.05).

To examine the effect of TGF-β1 on CTGF expression in LGSC cells, the human LGSC cell line MPSC1 was treated with 10 ng/mL TGF-β1 for different periods of time. As shown in Figure 1A, a 1 h treatment with TGF-β1 significantly up-regulated CTGF mRNA levels. The maximal effect was observed after 3 h of TGF-β1 treatment and then decreased but remained detectable after 24 h of treatment. In addition, the western blot results showed a similar effect of TGF-β1 on CTGF protein levels in MPSC1 cells (Figure 1B). Glycosylation of CTGF has been shown to produce multiple bands on a western blot [18]. Moreover, the stimulatory effect of TGF-β1 on CTGF expression was further confirmed in another human LGSC cell line, ILGSC (Figure 1C).

### SMAD3 signaling mediates TGF-β1-induced up-regulation of CTGF expression

**Figure 2:**
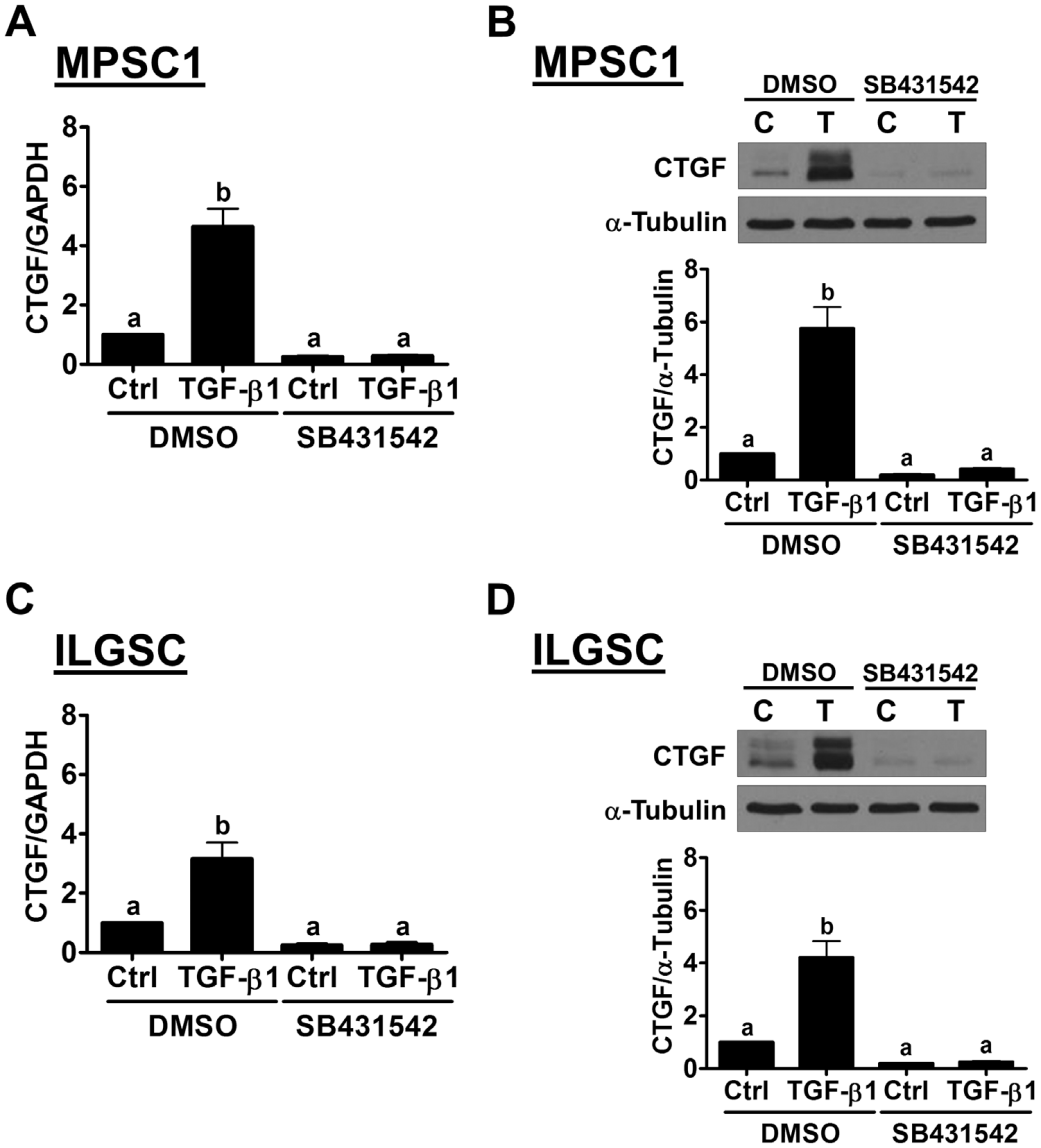
The TGF-β receptor is required for the TGF-β1-induced up-regulation of CTGF expression in LGSC cells **(A)** and **(B)**, MPSC1 cells were pretreated with vehicle control (DMSO) or 10 µM SB431542 for 1 h and then treated with vehicle control (Ctrl or C) or 10 ng/mL TGF-β1 (T) for 3 h. The mRNA A and protein B levels of CTGF were examined by RT-qPCR and western blot, respectively. **(C)** and **(D)**, ILGSC cells were pretreated with the vehicle control (DMSO) or 10 µM SB431542 for 1 h and then treated with vehicle control (Ctrl or C) or 10 ng/mL TGF-β1 (T) for 3 h. The mRNA A and protein B levels of CTGF were examined by RT-qPCR and western blot, respectively. The results are expressed as the mean ± SEM of at least three independent experiments. Values without a common letter were significantly different (*p*<0.05).

**Figure 3:**
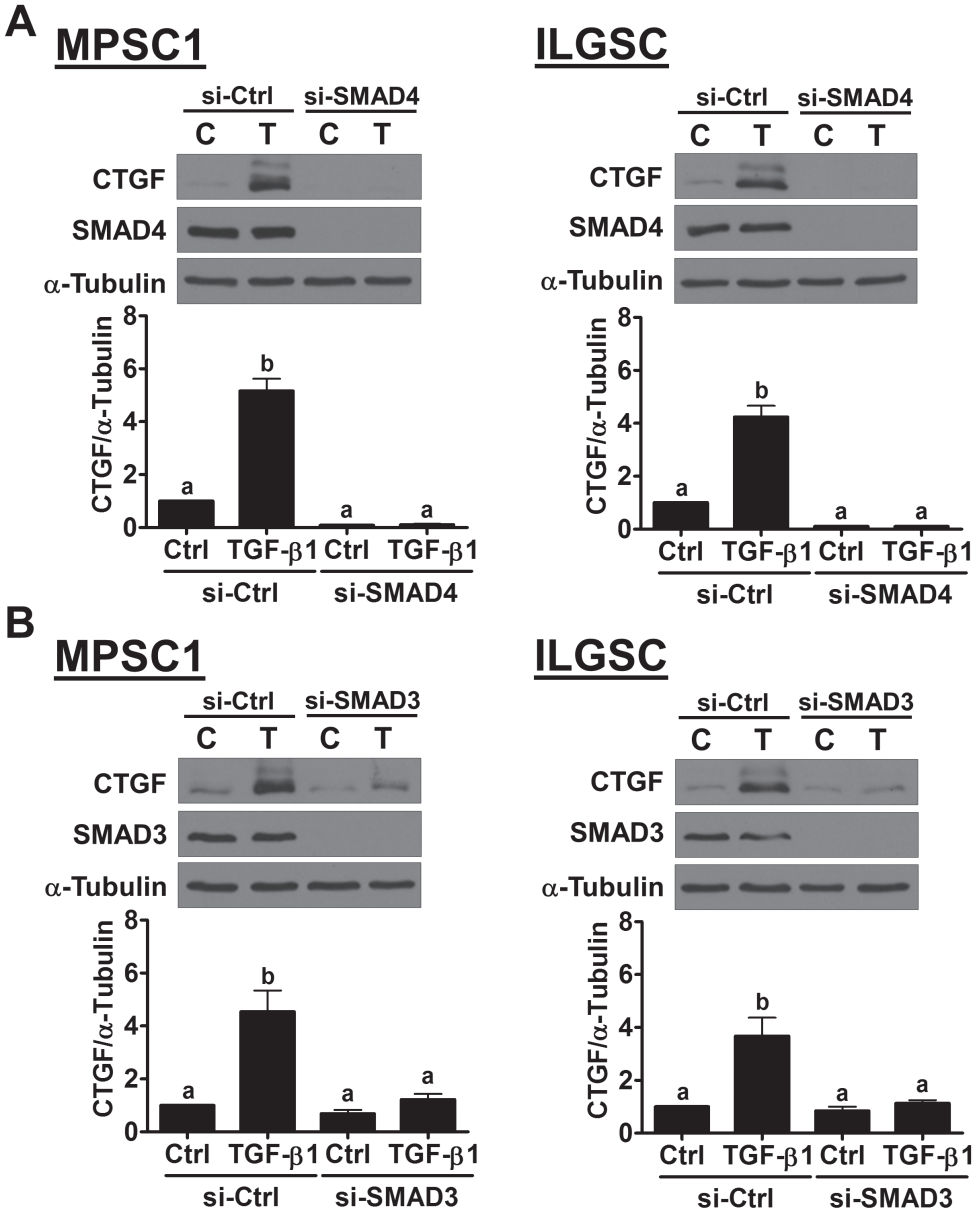
SMAD3 signaling is required for the TGF-β1-induced up-regulation of CTGF expression **(A)**, MPSC1 (left panel) and ILGSC (right panel) cells were transfected with 50 nM control siRNA (si-Ctrl) or SMAD4 siRNA (si-SMAD4) for 48 h and then treated with vehicle control (Ctrl or C) or 10 ng/mL TGF-β1 for 3 h. The protein levels of CTGF and SMAD4 were examined by western blot. **(B)**, MPSC1 (left panel) and ILGSC (right panel) cells were transfected with 50 nM control siRNA (si-Ctrl) or SMAD3 siRNA (si-SMAD3) for 48 h and then treated with vehicle control (Ctrl or C) or 10 ng/mL TGF-β1 for 3 h. The protein levels of CTGF and SMAD3 were examined by western blot. The results are expressed as the mean ± SEM of at least three independent experiments. Values without a common letter were significantly different (*p*<0.05).

It is well characterized that, upon binding of TGF-β1, the TGF-β type II receptor recruits and activates TGF-β type I receptor. The activated type I receptor then phosphorylates the downstream signaling molecules, SMAD2 and SMAD3. Phosphorylated SMAD2 and SMAD3, in turn, bind with the co-SMAD SMAD4, and the SMAD complexes can translocate to the nucleus, where they regulate gene expression [19]. To examine whether TGF-β receptor is required for TGF-β1-induced up-regulation of CTGF, a potent and specific TGF-β1 type I receptor inhibitor, SB431542, was used to block the signaling that was activated by TGF-β1 [20]. MPSC1 cells were pretreated with 10 μM SB431542 for 1 h and then treated with 10 ng/mL TGF-β1 for 3 h. The results of RT-qPCR showed that SB431542 abolished the TGF-β1-induced up-regulation of the CTGF mRNA levels without affecting its basal levels. The western blot results further confirmed the inhibitory effect of SB431542 on the TGF-β1-up-regulated CTGF protein levels in MPSC1 cells (Figure 2B). Similar results were observed in ILGSC cells (Figure 2C and 2D). To examine the involvement of SMAD signaling in TGF-β1-induced CTGF expression, the siRNA-mediated SMAD4 knockdown approach was used. As shown in Figure 3A, SMAD4 siRNA significantly down-regulated SMAD4 expression in both MPSC1 and ILGSC cells. In addition, knockdown of SMAD4 abolished TGF-β1-induced up-regulation of CTGF protein levels. Our previous study shows that TGF-β1 activates SMAD3, but not SMAD2, in MPSC1 cells [14]. Therefore, we knocked down SMAD3 to examine whether SMAD3 was required for TGF-β1-induced up-regulation of CTGF expression. As shown in Figure 3B, similar to SMAD4 knockdown, the knockdown of SMAD3 abolished TGF-β1-induced up-regulation of CTGF protein levels in both MPSC1 and ILGSC cells.

### Knockdown of CTGF attenuates TGF-β1-induced cell apoptosis by down-regulating SMAD3 expression

**Figure 4:**
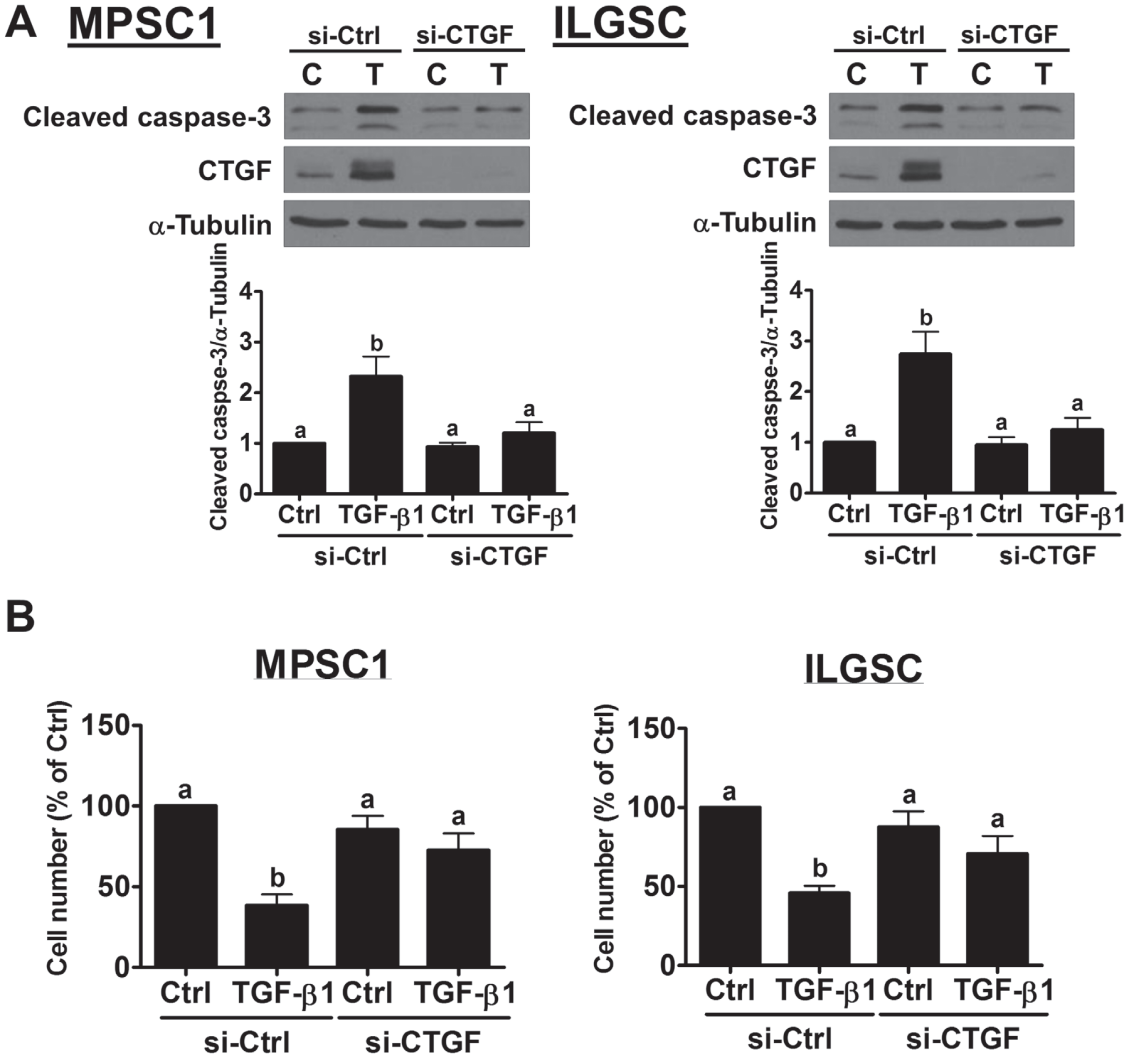
Knockdown of CTGF attenuates TGF-β1-induced cell apoptosis **(A)**, MPSC1 (left panel) and ILGSC (right panel) cells were transfected with 50 nM control siRNA (si-Ctrl) or CTGF siRNA (si-CTGF) for 48 h and then treated with vehicle control (Ctrl or C) or 10 ng/mL TGF-β1 for 24 h. The protein levels of cleaved caspase-3 were examined by western blot. **(B)**, MPSC1 (left panel) and ILGSC (right panel) cells were transfected with 50 nM control siRNA (si-Ctrl) or CTGF siRNA (si-CTGF) for 48 h and then treated with vehicle control (Ctrl or C) or 10 ng/mL TGF-β1 for 48 h. The cell number changes were examined by trypan blue exclusion assay. The results are expressed as the mean ± SEM of at least three independent experiments. Values without a common letter were significantly different (*p*<0.05).

**Figure 5:**
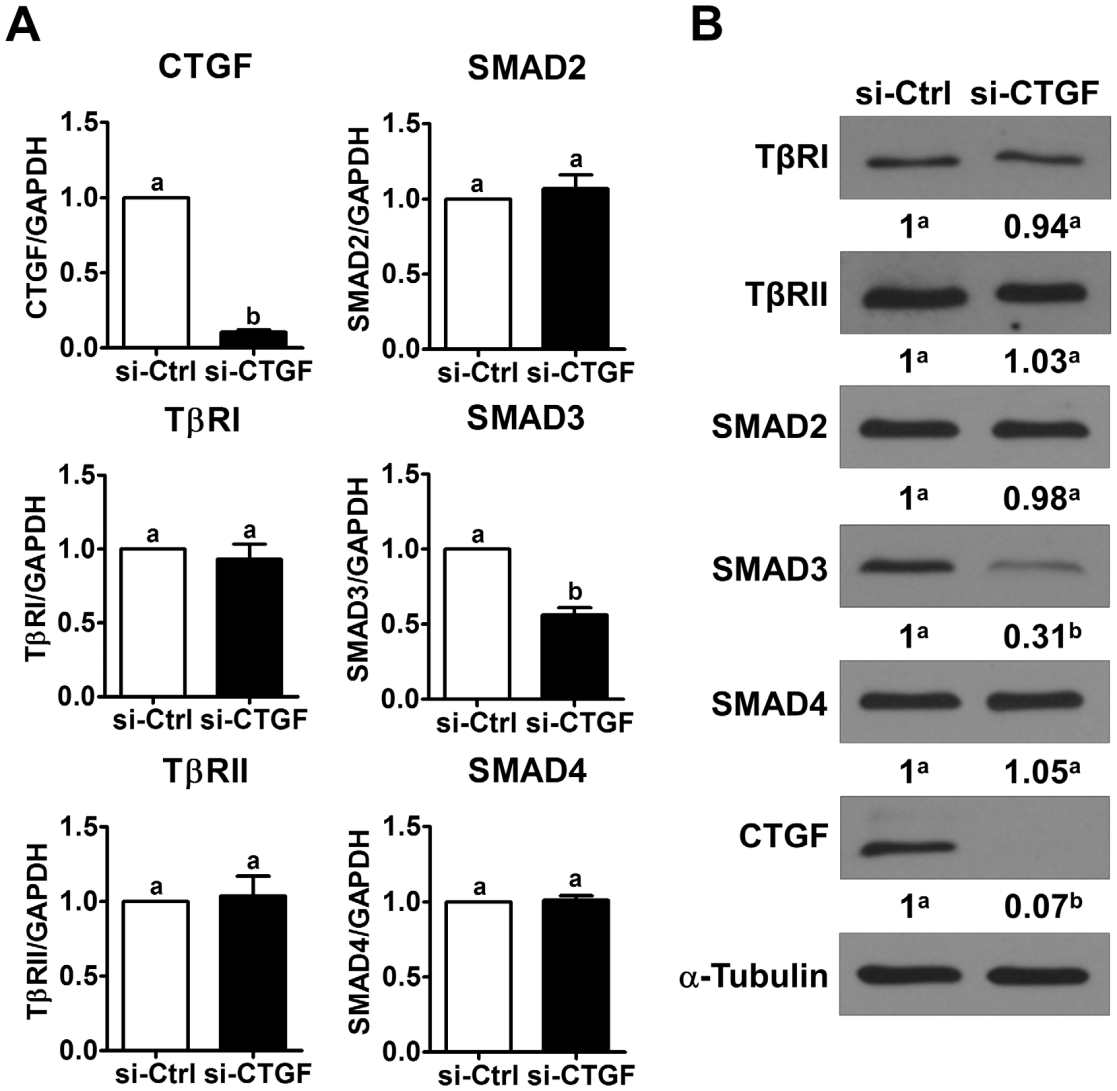
Knockdown of CTGF down-regulates SMAD3 expression **(A)**, MPSC1 cells were transfected with 50 nM control siRNA (si-Ctrl) or CTGF siRNA (si-CTGF) for 24 h. The mRNA levels of TGF-β receptor I (TβRI), TGF-β receptor II (TβRII), SMAD2, SMAD3 and SMAD4 were examined by RT-qPCR. **(B)**, MPSC1 cells were transfected with 50 nM control siRNA (si-Ctrl) or CTGF siRNA (si-CTGF) for 48 h. The protein levels of TGF-β receptor I (TβRI), TGF-β receptor II (TβRII), SMAD2, SMAD3 and SMAD4 were examined by western blot. The RT-qPCR results are expressed as the mean ± SEM of at least three independent experiments. Numbers under the western blots represent the densitometry quantifications. Values without a common letter were significantly different (*p*<0.05).

We have shown that treatment with TGF-β1 induces LGSC cells apoptosis [14]. To examine whether CTGF is involved in TGF-β1-induced LGSC cell apoptosis, CTGF siRNA was used to knockdown the expression of CTGF. As shown in Figure 4A, CTGF siRNA not only down-regulated the basal levels of CTGF but also abolished the TGF-β1-induced up-regulation of CTGF expression in both MPSC1 and ILGSC cells. Importantly, knockdown of CTGF attenuated TGF-β1-induced up-regulation of cleaved caspases-3. Consistent with our previous study, treatment with TGF-β1 decreased the cell number for both MPSC1 and ILGSC cells. The pro-apoptotic effect of TGF-β1 was attenuated by the knockdown of CTGF (Figure 4B). To examine the underlying mechanisms of the CTGF knockdown-attenuated effect of TGF-β1 in LGSC cells, MPSC1 cells were transfected with CTGF siRNA, and the expression levels of TGF-β1 signaling-related molecules were examined. As shown in Figure 5, knockdown of CTGF did not affect the mRNA and protein levels of TGF-β type I receptor, TGF-β type II receptor, SMAD2 and SMAD4. Interestingly, knockdown of CTGF down-regulated the mRNA and protein levels of SMAD3. These results indicated that knockdown of CTGF attenuated TGF-β1-induced cell apoptosis by down-regulating SMAD3 expression in LGSC cells.

## DISCUSSION

CTGF is widely expressed and has been shown to be an important modulator that regulates many physiological and pathological processes in a variety of tissues [18]. Aberrant expression of CTGF has been reported in many types of human cancer, and CTGF can function as a tumor promoter or suppressor in a context-dependent manner [21, 22]. To date, the role of CTGF in ovarian cancer has not been extensively studied. A previous study has shown that CTGF is expressed in normal OSE cells [23]. In ovarian cancer, the CTGF protein is differentially expressed among different histologic subtypes (serous, mucinous, clear cell and endometrioid). Interestingly, among all histologic subtypes, a lack of CTGF expression is found in the earlier stages (stage I and II) of disease, while the expression of CTGF is restored in the advanced stages (stage III and IV) and is associated with a more malignant phenotype. In addition, patients with stage I and II diseases with lower CTGF levels have a lower overall survival. Moreover, *in vitro* experiments reveal that CTGF can suppress cell proliferation in the clear cell and endometrioid subtypes of human ovarian cancer cells and ovarian granulosa cells [23, 24]. In contrast, previous studies have demonstrated that CTGF promotes cell migration and proliferation, and patients with higher CTGF expression levels are associated with poor survival in HGSC [11–13, 25]. Taken together, these results indicate that the role of CTGF in human ovarian cancer progression is complicated and its biological effects may depend on the subtype of the disease. LGSC is the least common type of human ovarian cancer. Compared to HGSC, LGSC is relatively slow growing. To the best of our knowledge, the expression and function of CTGF in LGSC are completely unknown. In the present study, our results showed that the expression of CTGF was detected by RT-qPCR and western blot in two human LGSC cell lines. Functionally, we showed that knockdown of CTGF did not affect basal cell proliferation but significantly attenuated TGF-β1-induced cell apoptosis. These results indicate that CTGF may act as a tumor suppressor in LGSC. Since LGSC is very rare, future investigations of whether CTGF expression levels correlate with clinical outcomes in LGSC will be of great interest.

CTGF can be regulated by many growth factors or cytokines through a variety of molecular mechanisms [5]. TGF-β1 has been shown to up-regulate CTGF expression, and this effect plays a key role in the development of fibrosis [3]. SMAD transcription factors are well-characterized downstream effectors of TGF-β1 that mediate TGF-β1-regulated gene expression [26]. A SMAD binding site on the CTGF promoter has been identified [27]. Our previous study has shown that TGF-β1 activates SMAD3, but not SMAD2, in LGSC cells [14]. In the present study, for the first time, we report that TGF-β1 can up-regulate CTGF expression in LGSC cells. In addition, knockdown of common SMAD4 or SMAD3 abolishes TGF-β1-up-regulated CTGF expression. Our results agree with a previous study showing that SMAD3, but not SMAD2, is involved in TGF-β1-up-regulated CTGF expression in other cell types [27–30]. Interestingly, in human granulosa cells, both SMAD2 and SMAD3 are required for the TGF-β1-induced up-regulation of CTGF expression [16]. In addition, SMAD signaling is not required for TGF-β1-induced up-regulation of CTGF expression in rat hepatic progenitor cells [31]. Taken together, these studies indicate that the specific SMAD protein required for TGF-β1-induced CTGF expression is cell-type dependent.

CTGF is able to affect the activity of many signaling pathways [18]. It has been shown that CTGF enhances the receptor binding of TGF-β1 by directly binding with TGF-β1. In contrast, the direct binding of CTGF and BMP4, a member of the TGF-β superfamily, prevents the receptor binding of BMP4 and hence antagonizes its function [32]. Treatment with recombinant CTGF does not directly activate SMAD signaling in human proximal tubule cells and cortical fibroblasts [33]. Interestingly, treatment with CTGF rapidly down-regulates the expression of SMAD7, a well-known key negative regulator of TGF-β1 signaling, and co-treatment with CTGF enhances TGF-β1-activated SMAD2/3 in human mesangial cells [34, 35]. Injection of TGF-β1 or CTGF individually does not induce persistent fibrosis in mice, whereas co-injection of TGF-β1 and CTGF induces sustained fibrosis [36]. These results indicate that CTGF can synergistically enhance the effects of TGF-β1. In the present study, we found that siRNA-mediated knockdown of CTGF down-regulated SMAD3 expression. However, whether CTGF knockdown affects SMAD7 expression in LGSC remains unknown and will be interesting for future studies. Nevertheless, our finding provides an alternative mechanism that CTGF is required for TGF-β1-regulated biological functions. Loss of CTGF can attenuate effects of TGF-β1 by decreasing SMAD3 expression, which consequently attenuates TGF-β1/SMAD3 signaling.

In summary, this study examined the role of CTGF in TGF-β-induced cell apoptosis in LGSC cells. Our results demonstrate that CTGF is up-regulated by TGF-β1 in two human LGSC cell lines. In addition, we show that activation of SMAD3 is required for TGF-β1-induced up-regulation of CTGF. Moreover, up-regulated CTGF is involved in TGF-β1-induced cell apoptosis. Knockdown of CTGF attenuates TGF-β1-induced cell apoptosis by down-regulating SMAD3 expression. These results suggest that CTGF may play important roles in LGSC progression.

## MATERIALS AND METHODS

### Cell culture

The MPSC1 cell line, which was established from an LGSC (provided by Dr. Ie-Ming Shih, Department of Pathology, Johns Hopkins Medical Institutions, Baltimore, MD), was maintained in RPMI 1640 (Invitrogen, Burlington, ON) supplemented with 10% fetal bovine serum (FBS; HyClone Laboratories Inc., Logan, UT) [37]. The SV40 LT/ST immortalized LGSC (ILGSC) cell lines were grown in a 1:1 (v/v) mixture of M199/MCDB105 medium (Sigma, Oakville, ON) supplemented with 10% FBS [38]. Cultures were maintained at 37°C in a humidified 5% CO_2_ atmosphere in air.

### Antibodies and reagents

Polyclonal anti-CTGF (#sc-14939) and monoclonal anti-α-tubulin (#sc-23948) antibodies were obtained from Santa Cruz Biotechnology (Santa Cruz, CA). Polyclonal anti-SMAD4 (#9515), anti-TGF-β receptor type I (#3712), anti-TGF-β receptor type II (#3713), anti-caspase-3 (#9662), monoclonal anti-SMAD2 (#3103) and anti-SMAD3 (#9523) antibodies were obtained from Cell Signaling Technology (Danvers, MA). Horseradish peroxidase-conjugated goat anti-mouse IgG and goat anti-rabbit IgG were obtained from Bio-Rad Laboratories (Hercules, CA). Horseradish peroxidase-conjugated donkey anti-goat IgG was obtained from Santa Cruz Biotechnology. Recombinant human TGF-β1 was obtained from R&D Systems (Minneapolis, MN). SB431542 was obtained from Sigma.

### Small interfering RNA (siRNA) transfection

To knock down endogenous SMAD3, SMAD4 or CTGF, cells were transfected with 50 nM ON-TARGET*plus* SMART*pool* SMAD3, SMAD4 or CTGF siRNA (Dharmacon, Lafayette, CO) using Lipofectamine RNAiMAX (Invitrogen, Burlington, ON). The siCONTROL non-targeting siRNA pool (Dharmacon) was used as a transfection control. The knockdown efficiency was examined by RT-qPCR or western blot analysis.

### Western blot

Cells were lysed in lysis buffer (Cell Signaling Technology), and the protein concentrations were determined using a DC protein assay kit with BSA as the standard (Bio-Rad Laboratories). Equal amounts of protein (50 µg) were separated by SDS polyacrylamide gel electrophoresis and were transferred to PVDF membranes. After being blocked with Tris-buffered saline (TBS) containing 5% non-fat dry milk for 1 h, the membranes were incubated overnight at 4°C with primary antibodies, followed by incubation with the HRP-conjugated secondary antibody. Immunoreactive bands were detected with an enhanced chemiluminescent substrate (Pierce, Rockford, IL) and X-ray film.

### Real-time quantitative PCR (RT-qPCR)

Total RNA was extracted using the TRIzol reagent (Invitrogen) according to the manufacturer's instructions. Reverse transcription was performed with 3 µg RNA, random primers and M-MLV reverse transcriptase (Promega, Madison, WI). The primers used for SYBR Green RT-qPCR were as follows: CTGF, 5'-GCG TGT GCA CCG CCA AAG AT-3' (sense) and 5'-CAG GGC TGG GCA GAC GAA CG-3' (antisense); TβRI, 5'-GTT AAG GCC AAA TAT CCC AAA CA-3' (sense) and 5'- ATA ATT TTA GCC ATT ACT CTC AAG G-3' (antisense); TβRII, 5'-TGT GGA TGA CCT GGC TAA CA-3' (sense) and 5'-TCG GTC TGC TTG AAG GAC TC-3' (antisense); SMAD2, 5'-GCC TTT ACA GCT TCT CTG AAC AA-3' (sense) and 5'-ATG TGG CAA TCC TTT TCG AT-3' (antisense); SMAD3, 5'-CCC CAG CAC ATA ATA ACT TGG-3' (sense) and 5'-AGG AGA TGG AGC ACC AGA AG-3' (antisense); SMAD4, 5'-TGG CCC AGG ATC AGT AGG T-3' (sense) and 5'-CAT CAA CAC CAA TTC CAG CA-3' (antisense) and GAPDH, 5′-GAG TCA ACG GAT TTG GTC GT-3′ (sense) and 5′-GAC AAG CTT CCC GTT CTC AG-3′ (antisense). RT-qPCR was performed using an Applied Biosystems 7300 Real-Time PCR System equipped with 96-well optical reaction plates. The specificity of each assay was validated by melting curve analysis and agarose gel electrophoresis of the PCR products. Assay performance was validated by assessing amplification efficiencies by means of calibration curves and ensuring that the plot of log input amount versus Δ Ct has a slope with an absolute value <0.1. At least three separate experiments were performed and each sample was assayed in triplicate. Water and mRNA without RT were used as negative controls. A mean value of the triplicates was used for the determination of relative mRNA levels by the comparative Ct method with GAPDH as the reference gene and using the formula 2^–ΔΔCt^.

### Statistical analysis

The results are presented as the mean ± SEM of at least three independent experiments. The results were analyzed by one-way ANOVA and Tukey’s multiple comparison test using the PRISM software. Significant differences were defined by values of *p* < 0.05.
